# Mechanisms associated with post-stroke depression and pharmacologic therapy

**DOI:** 10.3389/fneur.2023.1274709

**Published:** 2023-11-02

**Authors:** Qingyang Zhan, Fanyi Kong

**Affiliations:** ^1^Institute of Chinese Medicine, Heilongjiang University of Chinese Medicine, Harbin, China; ^2^Neurosurgery, Affiliated First Hospital, Harbin Medical University, Harbin, China

**Keywords:** stroke, depression, mechanisms, treatment, 5-HT, PSD, SSRIs, vitamin D

## Abstract

Stroke is one of the most common cerebrovascular diseases, which is the cause of long-term mental illness and physical disability, Post-stroke depression (PSD) is the most common neuropsychiatric complication after stroke, and its mechanisms are characterized by complexity, plurality, and diversity, which seriously affects the quality of survival and prognosis of patients. Studies have focused on and recognized neurotransmitter-based mechanisms and selective serotonin-reuptake inhibitors (SSRIs) can be used to treat PSD. Neuroinflammation, neuroendocrinology, neurotrophic factors, and the site of the stroke lesion may affect neurotransmitters. Thus the mechanisms of PSD have been increasingly studied. Pharmacological treatment mainly includes SSRIs, noradrenergic and specific serotonergic antidepressant (NaSSA), anti-inflammatory drugs, vitamin D, ect, which have been confirmed to have better efficacy by clinical studies. Currently, there is an increasing number of studies related to the mechanisms of PSD. However, the mechanisms and pharmacologic treatment of PSD is still unclear. In the future, in-depth research on the mechanisms and treatment of PSD is needed to provide a reference for the prevention and treatment of clinical PSD.

## 1. Introduction

A significant proportion of older adults will suffer from one or more age-related diseases, including two significant conditions that can cause high morbidity and disability, with high economic burden consequences: stroke (1) and depression (2). Stroke is divided into ischemic stroke and hemorrhagic stroke. The latter includes intracerebral hemorrhage (ICH) and subarachnoid hemorrhage (SAH). Depression is one of the most common symptoms of mental disorders after stroke, with a predicted prevalence of 18 to 33% (3–5). Depression will become the leading cause of global burden by 2030, so attention should be paid to treating depression (6, 7). PSD is mainly manifested as a lack of energy, apathy, sleep disorders, reduced interest, passivity, pessimism, and even suicide, and PSD quickly leads to stroke recurrence. Because many stroke patients have cognitive and language disorders, PSD is not easy to find but seriously hinders the rehabilitation of patients. PSD is a crucial risk factor for long-term adverse physical and mental health outcomes after stroke (8), and PSD can lead to lower quality of life and higher mortality (9). Several stroke-related psycho-social factors worsen, at best, the PSD symptoms by negatively impacting the patient’s daily life (10–12). Much literature has focused on the mechanism of post-ischemic depression, and its mechanisms has become a research hotspot in recent years. Given this, it is necessary to understand the mechanisms and drug treatment of PSD, which can provide a reference for the prevention and treatment of clinical PSD.

## 2. Stroke lesion site and PSD

Soares proposed a neuroanatomical model of mood disorders in 1997, i.e., the pathway of emotion regulation includes the frontal lobe, the basal ganglia, the amygdala-hippocampus complex, the thalamus, and the connecting fibers between them (13). The frontal-subcortical neural pathway regulates sleep, mood, neuroendocrine, motor and cognitive behaviors, etc. The basal ganglia is an essential pathway for the axons of 5-HT and DA neurons, and the above ways and lesions block the axonal transmission of neurotransmitters to the cortex. Hence, lesions of the above areas are prone to depression. The central monoaminergic nuclei are located in the brainstem, and they fire ascending projections distributed throughout the brain, including the cerebral cortex and limbic system. It is thought that ischemic damage to these nuclei or their projections may result in decreased monoamine levels in a) the left frontal cortex, leading to depressed mood and cognitive deficits; b) the reward system, leading to a lack of pleasure; and c) the basal ganglia, which directly and indirectly regulate mood, cognition, reward, and fatigue (14, 15). The occurrence of stroke in specific regions such as the prefrontal cortex, limbic area, and basal ganglia can disrupt key pathways of mood-related neurotransmitters, leading to depressive disorders (16, 17). Studies have shown that PSD is strongly associated with the site of stroke lesions, but different studies have different results. Terroni et al. (18) further affirmed that the limbic-cortical-striatal-pallidal-thalamic neural pathway is closely associated with PSD based on previous imaging studies. In the 1980s, Robinson et al. suggested that injury to the anterior part of the left hemisphere is more likely than other body parts to lead to depression and PSD (19, 20). Injury to the left cerebral hemisphere is associated with depression, especially damage to the left frontal cortex and the left basal ganglia has a higher incidence of depression, and 5-HT and NE in the left cerebral hemisphere are more likely to be depleted than those in the right cerebral hemisphere, and damage to NE and 5-HTergic neuronal pathways in the above regions, which decreases the content of NE and 5-HT and thus leads to PSD (21–23). However, one study did not support the hypothesis that left hemisphere lesions are associated with an increased risk of PSD, and there was a significant correlation between right hemisphere stroke and the risk of depression after subacute stroke (1–6 months) (24). A significant association between damage to the subcortical circuit in the frontal lobe and PSD has been found (25). In addition, Hong et al. (26) collected 23 patients with PSD diagnosed with frontal subcortical onset. The gray matter volume of the left middle frontal gyrus was significantly reduced in the PSD patients compared with the non-PSD group. The lesion site was located in the left inferior frontal gyrus in about 14 PSD patients, and the lesion site was found in the dorsolateral prefrontal cortex in about 9 PSD patients. Leukoaraiosis also called white matter lesions (WMLs) and white matter hyperintensities (WMHs). Multiple studies have shown that deep leukorariosis with PSD (27). Determining the extent of pre-existing white matter abnormalities can properly guide decision making in acute stroke settings, as a greater degree of such lesioning is usually coupled with neuropsychiatric aftermaths, such as PSD (28). Although there is no uniform conclusion on whether lesion site is associated with PSD, most researchers still believe there is a relationship between lesion site and the occurrence of PSD.

## 3. Neurotransmitter hypothesis

Monoamine neurotransmitters mainly include norepinephrine (NE), 5-hydroxytryptamine (5-HT), and dopamine (DA), and most of their receptors belong to the G-protein-coupled receptor. NE, 5-HT, and DA transmit messages between nerve cells or neurons and effector cells, integrating the overall coordination of body functions. If these neurotransmitters are defective, the normal functioning of the nervous system is compromised, leading to depression (29, 30). Ischemic injury interferes with upward projections from the midbrain and brainstem, reducing the bioavailability of 5-HT, DA, and NE (31). Many neurophysiological studies found an early involvement of the central serotonergic tone since the very acute phase of stroke and in all stroke patients as a group, regardless the degree of disability and the site of the lesion (32–34). In the presence of the SLC6A4 linked promoter region (5-HTTLPR) s/s genotype promoter methylation status was independently associated with PSD both at 2 weeks and more prominently at 1 year after stroke, and was significantly associated with the worsening of depressive symptoms over 1 year (35). Previous studies have demonstrated that 5-hydroxytryptamine transporter length polymorphism (5-HTTLPR) predicts stress and depression (36). Wang et al. (37) successfully prepared a PSD model and found that depressive symptoms in rats could be blocked by the SSRIs citalopram or the 5-HT1A receptor blocker WAY-100635, and detected an increase in newborn neurons in the hippocampal DG region, suggesting that SSRIs act by promoting neural regeneration in the hippocampal DG region. Mak et al. conducted a meta-analysis and found that the 5-HTTLPR LL, LS, and LS genotypes, and the L allele had a positive effect on PSD recovery, but the SS gene in 5-HTTLPR may be a risk factor for PSD (38, 39). Therefore, monoamine neurotransmitters and genes are one of the mechanisms most closely associated with PSD.

### 3.1. Amino acid neurotransmitters and PSD

After a stroke, acute ischemia/hypoxia occurs in brain tissue, leading to ion transporter dysfunction and ion homeostasis disturbances, which in turn leads to impaired glutamate release and reuptake and intracellular calcium overload, which further contributes to the rapid rise in cerebrospinal fluid glutamate levels. These cascading reactions ultimately lead to neuronal death (40, 41). In addition, excessive glutamate release may lead to synaptic excitotoxicity by exacerbating oxidative stress and inflammation. In contrast, inflammatory mediators may interfere extracellular glutamate levels by decreasing the glutamate scavenging capacity of microglia and astrocytes (42, 43). Many studies have reported higher levels of glutamate and its metabolites in both the blood and cerebrospinal fluid of patients with PSD, especially in the frontal cortex (44, 45). The above experiments suggest that amino acid neurotransmitters are equally involved in developing PSD.

## 4. Neuroinflammation

In addition to classical neurotransmitters, astrocytes and microglia in the central nervous system induce cytokine production, including interleukin (IL), tumor necrosis factor (TNF), and interferon (IFN). When the body undergoes an inflammatory response induces the expression of relevant inflammatory cytokines, and an increase in inflammatory cytokines leads to a decrease in the amount of 5-HT or even depletion. Serum inflammatory cytokine levels are elevated in patients with depression, and antidepressant drugs, such as SSRIs, can decrease the pro-inflammatory cytokines IL-6, IL-1β, TNF-α, and IFN-γ, or increase the anti-inflammatory cytokines, such as IL-10, IL-4, IL-13 (46, 47). Inflammation triggers depression by affecting the normal secretion and synthesis of monoamine neurotransmitters, neuronal regeneration, and stimulation of glial cell activation in various ways. Spalletta et al. investigated and put forward the “cytokine hypothesis,” in which pro-inflammatory cytokines interact with 5-HT, leading to the amplification of inflammatory processes and activation of indoleamine-2,3-dioxygenase (IDO) in the limbic region (48). Activation of IDO in the limbic region converts tryptophan to kynurenine, leading to depletion of 5-HT in the paralimbic structures, and the resulting physiological dysfunction may lead to PSD. Elevated levels of inflammatory mediators are thought to be associated with PSD, and increases in pro-inflammatory cytokines IL-1, IL-2, IL-6, IL-17, IL-1β, and TNF-α are strongly associated with PSD (49–51). In another study, a total of 151 patients with acute ischemic stroke were screened at baseline and completed a 1-month follow-up, serum IL-10 levels were measured within 24 h of admission, and depressive symptoms were assessed using the 17-item Hamilton Depression Scale (HAMD-17), with PSD defined as a HAMD score of ≥7. It was found that serum levels of IL-10 were significantly lower in patients with PSD than those in the non-PSD group (52). Alleles associated with reduced anti-inflammatory cytokine function, such as IL-4 + 33C/C and IL-10-1082A/A genotypes, were also associated with PSD (53). In addition, microglia can be distinguished into two phenotypes, the deleterious pro-inflammatory M1 type and the anti-inflammatory M2 type, which represent the dual role of microglia. M1 microglia promote the release of a range of pro-inflammatory cytokines such as TNF-α, IL-1-β, IL-6, and nitric oxide (NO), as well as protein hydrolyzing enzymes, such as matrix metalloproteinase-9 (MMP-9) and MMP-2, which ultimately exacerbate neuronal injury and inhibit neurogenesis in the hippocampus. M2-type microglia express the protective cytokines CD206, IL-10, and scavenger receptors, which have a role in inhibiting inflammation and promoting tissue repair.

NLRP3 inflammatory vesicle is a multiprotein complex of the natural immune system and an upstream regulator of IL-1β. Activation of NLRP3 inflammatory vesicle activates cysteine aspartate lyase-1 via NF-κB and MAPK pathways, induces IL-1β and IL-18 production, and thus promotes inflammatory responses (54, 55). It has been found that lack of NLRP3 attenuates LPS-induced depressive-like symptoms and increases IDO gene expression while inhibiting microglia activation, suggesting that IDO may be a downstream mediator of NLRP3 inflammatory vesicles in inflammation-mediated depressive-like behavior (56). Therefore, Li et al. proposed lowering NLRP3 levels as a treatment for PSD.

Serum growth differentiation factor-15 is a transforming growth factor-β (TGF-β)-related cytokine (57). High levels of serum growth differentiation factor-15 are significantly associated with poor clinical outcomes in acute ischemic stroke, suggesting that serum growth differentiation factor-15 levels can predict the prognosis of ischemic stroke patients (58). High serum growth differentiation factor-15 levels may be associated with an increased risk of suicidal thoughts in depressed patients (59). Many recent studies have pointed out that serum growth differentiation factor-15 levels are nearly one-fold higher in patients with PSD than in patients without depression, and the sensitivity and specificity for predicting PSD were most heightened when the level was 1,660 ng/L (60, 61). MMP-9 is a crucial determinant of extracellular matrix degradation, which is involved in inflammatory response and neuronal plasticity, and it plays a role in the development of brain injury and depression. Elevated serum MMP-9 levels during the acute phase of ischemic stroke were found to be closely associated with the development of depression 3 months later (62). Hypersensitive C-reactive protein (Hs-CRP) is closely related to neurological injury in the acute phase of stroke. It can be used as a serum inflammatory indicator reflecting the intensity of inflammation in the body (63). Hs-CRP can be used as a diagnostic marker for depression, especially for male patients with depression (64). Some studies have found that elevated serum CRP levels on admission are associated with an increased risk of PSD (65). Homocysteine (Hcy) can be used as a biomarker of stroke, and along with abnormally elevated Hcy levels, methylation metabolism is blocked, and NE and 5-HT levels are reduced, thus leading to depression (66, 67). And it has been found that high levels of Hs-CRP and higher Hcy in ischemic stroke patients may also be associated with PSD (68, 69).

In addition to the well-recognized microglia, other inflammatory markers have attracted widespread attention. A meta-analysis examined whether neutrophil-tolymphocyte ratio (NLR) platelet-lympho-cyte ratio (PLR) and monocyte-to-lymphocyte (MLR) were associated with depression and found that NLR levels were significantly higher in depressed patients than in healthy controls (70). Two hundred and ninety-nine consecutive ischemic stroke patients were enrolled and followed up for 1 month; 26.1% of patients were diagnosed with PSD at 1 month, and patients with PSD had significantly higher NLR levels on admission compared with non-PSD patients and normal controls, with an NLR ≥3.701 independently associated with the development of PSD (71). A recent meta-analysis showed that higher inflammation ratios, especially NLR, were significantly associated with the risk of developing depression and that compared to non-PSD patients, PSD patients had a significantly higher NLR and MLR values were higher in PSD patients (72). Higher platelet count is a predictor of inflammation, and platelet activation and increased platelet counts play an important role in depression, as well as being one of the risk factors for the increased prevalence of cerebrovascular disease, and patients with major depression with psychotic features have a higher PLR than other patients (73). Elevated PLR on admission is an important and independent marker for predicting the development of PSD, and whether it changes over time remains to be thoroughly investigated (74). Sarejloo et al. (75) found that the NLR was higher in patients with PSD than in non-depressed patients with stroke, and the PLR was significantly higher in patients with PSD than in non-depressed patients with stroke.

## 5. Neuroendocrine

The HPA axis is the neuroendocrine system that regulates mood; first, when the hypothalamus receives signals from the hippocampus or other tissues, the paraventricular nucleus of the hypothalamus releases corticotropin-releasing hormone (CRH), which induces adrenal cortical hormone (ACTH) and glucocorticoid (GCs).

Adrenocorticotropic Hormone (ACTH) stimulates the synthesis and secretion of GCs in the zona fasciculate by binding to its primary target. As downstream effectors of the HPA axis, GCs enter the circulation and readily cross the blood–brain barrier to regulate physiological changes via intracellular receptors throughout the body. Elevated cortisol concentrations in plasma, urine, and cerebrospinal fluid have been reported in depressed patients, accompanied by downregulation of peripheral 5-HT, hyperactivation of the HPA axis, and upregulation of ACTH (76, 77). Compounds with GC receptor antagonist activity and 5-HT1A receptor agonist activity may be better drugs for treating depression (78, 79). HPA dysfunction is present in 40% of stroke patients, triggering depression, poor prognosis, and increased death are associated (80). Excess cortisol may also be associated with monoamine dysfunction, and in a recent clinical study, Reimold et al. investigated the correlation between cortisol response and thalamic 5-HT transporter levels using positron emission tomography and found that decreased levels of thalamic 5-HT transporters were significantly correlated with elevated cortisol response (81).

## 6. Neurotrophic factor

Brain Derived Neurotrophic Factor (BDNF) has a variety of biological functions; through the activation of tropomyosin receptor kinase B (TrkB) receptor and p75NTR receptors, TrkB and p75NTR pathway activation lead to opposed effects, BDNF requires signaling through TrkB in neuronal growth and maturation. In contrast, the p75NTR pathway triggers apoptosis and inhibits synapse formation. Activation of the TrkB and p75NTR pathways leads to opposed effects, with BDNF required to signaling through TrkB in neuronal growth and maturation. In contrast, the p75NTR pathway triggers apoptosis and inhibits synapse formation, and BDNF is involved in the physiological and pathological processes of depression and ischemic stroke (82, 83). Yang et al. established a PSD model by oxygen–glucose deprivation and corticosterone treatment of neuronal cells. proBDNF protein levels were significantly elevated in the cortex and hippocampus of rats in the PSD group compared to the control group, suggesting that proBDNF plays a role in PSD pathophysiology (84). In addition, a PSD-like cell model was re-established by recombination of the p75 neurotrophin receptor (p75NTR) or silencing of the c-Jun amino-terminal kinase (JNK) to re-establish a PSD-like cell model and found that p75NTR and silencing of JNK (siJNK) inhibited PSD-induced proBDNF up-regulation and increased apoptosis (84). In the same cohort, higher BDNF methylation status and BDNF val66met polymorphism were independently associated with the prevalence of PSD (85).

Glial Cell Line-derived Neurotrophic Factor (GDNF) is widely distributed in the hypothalamus and other brain parts. The role of GDNF in the brain is essential in the survival, differentiation, and regeneration of neurons in the ischemic hemiparetic zone. GDNF can protect 5-HT and DA neurons from oxidative stress and neuroinflammatory damage and has neurotrophic effects on brain tissue (86). Lower levels of GDNF may be involved in the pathophysiological processes of depression, and GDNF levels increase after antidepressant treatment (87). Some scholars have found that GDNF and mRNA are closely related to PSD, and GDNF can be used as a biomarker for the differential diagnosis of major depression and PSD (88). It further suggests that GDNF may act on neurotransmitters and thus participate in the development of PSD.

IGF-1 has received much attention for its influence on recovery after stroke (89). Ketamine, an n-methyl-d-aspartate receptor antagonist, exerts antidepressant effects, and ketamine also induces sustained massive release of IGF-1 in the prefrontal cortex of male mice (90). A clinical trial by Wei Zhang et al. suggested that low serum IGF-1 levels on admission may be involved in developing PSD (91). Recent studies suggest that carriers of the T allele at the rs9282715 locus of the IGF-1R gene may be susceptible to PSD (92).

## 7. Pharmacological treatment of post-stroke depression

Antidepressant medicines can effectively improve patients’ nxiety, depression, and somatization symptoms, the first choice for PSD treatment. The principle of medication is to use the smallest effective dose possible to minimize the adverse effects and improve adherence to the treatment. In the early days, antidepressants such as TCAs, tetracyclines, and monoamine oxidase inhibitors were the mainstay, but these antidepressants had more side effects. SSRIs gradually replaced them with fewer adverse effects and better-tolerated drugs such as NaSSA.

### 7.1. SSRIs

SSRIs, a new class of antidepressant drugs used in clinical applications, began in the 1980s. SSRIs are mainly fluoxetine, paroxetine, sertraline, citalopram, and escitalopram, which can selectively inhibit the presynaptic membrane to the reuptake of 5-HT (93). Studies have demonstrated the efficacy, acceptability, and tolerability of antidepressant medication in patients with PSD (94). There have been small-sample controlled clinical studies showing that fluoxetine (95, 96) and citalopram (96) are effective in the treatment of PSD. Fluoxetine treatment promotes microglial apoptosis (97). Early use of SSRIs such as escitalopram may be a treatment for PSD (98, 99). Zhang et al. (100) retrieved Meta-analyzes up to December 2021. They found that antidepressant co-adjuvant therapy may enhance the efficacy of antidepressant medications, with acupuncture combined with fluoxetine being more efficacious in treating PSD at week 4. In contrast, rTMS combined with paroxetine was more productive in treating PSD at week 8 and was more efficacious. It has been suggested that SSRI treatment may be beneficial in stroke patients to prevent the development of PSD, but treating all stroke patients with SSRI has not proved effective so far. Large, prospective and long-term studies are needed to clarify the possible impact of SSRIs on emotions, cognitive functions, bone fractures and coagulation, as well to detect other possible still neglected side effects (101). Thus, assessing the central serotonergic tone in acute stroke patients through auditory evoked potentials may also help to predict the responsiveness to the SSRI treatment and individuate the subgroup of PSD patients who may benefit from SSRI treatment (102–104).

### 7.2. NaSSA

The representative drug of NaSSA is mirtazapine, which increases the release of 5-HT by directly inhibiting the α2 receptors at the endings of 5-HT neurons and also stimulates the α2 receptors on the cytosolic bodies of 5-HT neurons by increasing the NE content to increase the release of 5-HT further. Li et al. the eighth week of administration of the drug, Mirtazapine may be the best choice for treating PSD patients compared to other antidepressants (105). A study found that ischemic stroke patients who received 30 mg of mirtazapine or no antidepressant treatment starting on day 1 after stroke developed PSD in 40% of the untreated group. In contrast, PSD occurred in only 5.7% of the patients in the group treated with mirtazapine, with 16 patients experiencing PSD, of which 15 resolved after initiating mirtazapine treatment (106).

### 7.3. Anti-inflammatory drugs

Anti-inflammatory drugs can increase the concentration of monoaminergic neurotransmitters in the synaptic gap of the neurons involved in the brain in a short period. It has been found that the use of acetylsalicylic acid (ASA), nonsteroidal anti-inflammatory drugs (NSAIDs), or statins in stroke patients reduced the risk of early-onset depression, but a higher risk for late depression (107). Minocycline is widely recognized as a novel agent capable of inhibiting microglial activation (108), It also exerts anti-inflammatory properties (109). A systematic review revealed that minocycline increases the viability of neurons and decreases the infarct volume following cerebral ischemia, the mechanisms included anti-inflammatory, antioxidant, as well as anti-apoptotic effects (110). Bassett et al. (111) studies have found that minocycline reverses the pathogenic phagocytic potential of neurotoxic M1 microglia, and reduces the negative phenotypes associated with reduced neurogenesis caused by mice exposure to chronic mild stress (CMS) induced depressive-like behavior. Camargos et al. (112) used the clamping of the common carotid arteries bilaterally in C57BL/6 mice to prepare a cerebral ischemia–reperfusion injury model, and minocycline improved depression-like behavior in cerebral ischemia mice.

### 7.4. Vitamin D

Vitamin D is the only neurosteroid hormone that may regulate 5-HT synthesis via tryptophan hydroxylase 2 (113). Vitamin D may affect the synthesis of neurotransmitters, such as serotonin and dopamine, and is also involved in changes in brain morphology (114, 115). Pertile et al. (114) study continues to establish vitamin D as an important differentiation agent for developing dopamine neurons, and now for the first time shows chronic exposure to the active vitamin D hormone increases the capacity of developing neurons to release dopamine. According to previous research, vitamin D deficiency may be a risk factor for depression (116, 117). A prospective study encompassed 58,646 healthy Japanese adults (23,099 men and 35,547 women) aged of 40 to 79 years in whom dietary vitamin D intake was determined via a self-administered food frequency questionnaire. The median follow-up period was 19.3 years (1989–2009), and dietary vitamin D intake appeared to be negatively correlated with mortality from stroke (118). A Meta-analysis conducted by Zhou et al. (119) found that lower vitamin D levels were associated with an increased risk of ischemic stroke. Berghout et al. (120) conducted a prospective study measuring serum 25-hydroxyvitamin D concentrations in 9,680 participants (56.8% female) aged ≥45 years from 1997 to 2008, and lower serum 25-hydroxyvitamin D concentrations were not associated with a higher risk of stroke. It was not associated with a higher risk of stroke, and only severe vitamin D deficiency was associated with incident stroke. Gu et al. found a higher prevalence of vitamin D deficiency and insufficiency in patients with acute stroke. Low serum vitamin D levels were associated with the development of PSD (121, 122). Another study found that 55 (29.1%) patients with acute ischemic stroke were diagnosed with PSD at 1 month, and lower serum vitamin D within 24 h of admission was associated with PSD development and predicted PSD at 1 month (123). The stimulatory effects of vitamin D3 on the BDNF signaling pathway and neuroplasticity may play a role in the recovery of neurological function and the amelioration of PSD (124).

## 8. Summary

Multiple mechanisms are interrelated and interact in PSD. Monoamine neurotransmitters are the most important pathogenetic mechanism in PSD, and inflammatory cytokines and microglia can cause a decrease in 5-HT in the brain; altered ratios of monocytes, neutrophils, and lymphocytes can be used as a predictive biomarker for PSD, and there is a correlation between higher proinflammatory factors, NLRP3, TGF-β, Hs-CRP, Hcy with PSD, and the PSD interconnection between neurotrophic factor, neuroendocrine abnormalities, and The interconnections between stroke lesion sites and neurotransmitters involve multiple systems in the body thereby inducing PSD. Currently, PSD is mainly treated with SSRIs, NASSA, anti-inflammatory drugs, and medications such as vitamin D. In the future, many studies are needed to find more critical mechanisms for clinical reference, with the aim of early prediction of PSD and intervention to reduce the morbidity and mortality of PSD.

## Author contributions

Q-YZ: Formal analysis, Resources, Writing – original draft, Writing – review & editing. F-YK: Formal analysis, Methodology, Supervision, Validation, Writing – original draft, Writing – review & editing.
